# Stability and change in fruit and vegetable intake of Brazilian adolescents over a 3-year period: 1993 Pelotas Birth Cohort

**DOI:** 10.1017/S1368980015001664

**Published:** 2015-06-03

**Authors:** Romina Buffarini, Ludmila C Muniz, Aluísio JD Barros, Cora L Araújo, Helen Gonçalves, Ana MB Menezes, Maria CF Assunção

**Affiliations:** 1 Post-Graduate Program in Epidemiology, Federal University of Pelotas, CP 464, 96001-970 Pelotas, RS, Brazil; 2 Department of Nutrition, School of Nutrition, Federal University of Pelotas, Pelotas, RS, Brazil

**Keywords:** Fruits and vegetables, Tracking, Adolescents, Cohort studies

## Abstract

**Objective:**

To assess the stability and changes in fruit and vegetable (FV) consumption over a 3-year period during adolescence in a population-based birth cohort.

**Design:**

Longitudinal descriptive study. FV consumption was collected in 2008 and 2011/12 using an FFQ. We conducted descriptive analyses of medians to assess the trends in FV intake over time. Stability of FV intake was assessed by percentage of agreement and kappa coefficients.

**Setting:**

Pelotas, Rio Grande do Sul, Brazil.

**Subjects:**

Adolescents from 15 to 18 years of age (*n* 3915).

**Results:**

We observed an overall slight decrease in FV consumption during adolescence and also a moderate stability, especially in those with higher socio-economic status (proportion of agreement 38·6 % and 40·5 % for boys and girls, respectively). About a half of those consuming low levels of FV at 15 years of age still consumed low levels 3 years later.

**Conclusions:**

Our results showed that FV consumption presented a moderate stability across a 3-year period during adolescence, especially in those with higher socio-economic status. Given the great proportions of non-communicable diseases such as CVD, diabetes and obesity, knowledge about the patterns of FV consumption during adolescence has implications for health promotion interventions.

Fruits and vegetables (FV) are important components of a healthy diet. They usually have low energy density and are sources of many vitamins, minerals and other bioactive components that bring health benefits^(^ ^1^ ^–^ ^3^ ^)^. According to WHO, low consumption of FV is one of the top ten risk factors for global mortality. Their adequate intake reduces the risk of non-communicable chronic diseases and also prevents and corrects multiple micronutrient deficiencies, especially in low- and middle-income countries^(^ ^1^ ^,^ ^2^ ^,^ ^4^ ^)^.

Despite existing strategies and campaigns such as the ‘global strategy of food, physical activity and health’ and ‘5 a day’ which aim to increase the consumption of FV^(^ ^5^ ^,^ ^6^ ^)^, FV intake is still low among individuals of all ages in comparison with the current recommendation (400 g/d)^(^ ^6^ ^–^ ^8^ ^)^. Furthermore, several studies have found that this scenario of low consumption is especially common among children and adolescents from low-, middle- and high-income countries^(^ ^9^ ^–^ ^11^ ^)^. For instance, the literature shows that many Brazilian adolescents do not eat enough FV^(^ ^12^ ^–^ ^14^ ^)^.

There is growing evidence that major behavioural and biological risk factors arise early in life and continue to have a negative impact over the subsequent years^(^ ^1^ ^)^. In addition, studies assessing eating habits suggest there are critical aspects during adolescence that have an impact on chronic diseases, such as the development of risk factors during this period and their potential stability throughout life^(^ ^1^ ^,^ ^15^ ^–^ ^17^ ^)^. The latter could be measured through tracking analyses, which assess the stability of a certain outcome variable over a period of time. These analyses involve the maintenance of a relative position within a distribution of values over time^(^ ^18^ ^–^ ^20^ ^)^. Also, the study of the dynamics of consumption towards a less or more healthy direction may be important in predicting the development of a disease.

In high-income countries, there are several studies reporting longitudinal trends of consumption of FV^(^ ^15^ ^,^ ^21^ ^–^ ^28^ ^)^. However, we could not find studies assessing this item among low- and middle-income countries, where this information has become relevant for the nutrition transition phase that is taking place in those settings^(^ ^1^ ^)^.

Therefore, the purpose of the present study was to examine the stability and changes in FV intake of urban Brazilians adolescents over a 3-year period. A greater knowledge of these issues will be useful for the development of actions to promote healthful eating habits during this life stage.

## Methods

### Study design and population

Pelotas is a middle-sized city in southern Brazil with nearly 330 000 inhabitants. In 1993, all hospital-delivered newborns (99 % of all births) resident in the urban area of the city were eligible for the study; there were only sixteen refusals and information was obtained on 5249 live births. Since then, the cohort members have been interviewed at different time points. Further details of the methodology have been published previously^(^ ^29^ ^,^ ^30^ ^)^.

The present study included data from two visits, the 15- and 18-year follow-ups (carried out in 2008 and 2011/12, respectively), in which all cohort members were sought. The 2011/12 follow-up is referred to hereafter as the ‘2011 follow-up’. The response rate was 85·2 % and 81·4 %, respectively.

### Assessment of dietary intake

In the 15-year follow-up, the participants were asked to complete an eighty-one-item FFQ to assess dietary intake. The reproducibility and validity of this questionnaire have been described previously by Sichieri and Everhart^(^ ^31^ ^)^. The frequency of consumption over the preceding year was evaluating by the mean number of times (zero to ten) each food was consumed per day, week, month or year. To construct the FV intake variable, all fruits and vegetables items were summed. This variable was standardized into day frequencies in order to obtain a single time unit.

In the 18-year follow-up, a self-administered and semi-quantitative FFQ was applied which included eighty-eight items with a recall period of a year. Response scales were adapted from the proposal made by Willett with some modifications^(^ ^32^ ^)^. Possible answers for every item were: ‘never or less than one a month’, ‘one to three times a month’, ‘once a week’, ‘two to four times a week’, ‘five to six times a week’, ‘one time a day’, ‘two to four times a day’ and ‘five or more times a day’ (coded as 0, 0·067, 0·143, 0·429, 0·786, 1, 3 and 5 times/d, respectively). Finally, all the items were summed to create the daily FV intake variable.

In both FFQ the same twenty-four items of fruits and vegetables (excluding fruit juice) were considered. So, the daily frequency of FV intake indicates the total number of times per day that the adolescent ate any fruit or vegetable. When adolescents mentioned intake of an item only during the harvest period (seasonality), it was decided to divide this consumption by four, in order to represent the three months of the ‘harvest season’.

### Outcome variable

Our outcome variable was the daily frequency of FV consumption, assessed as a continuous variable and then divided into quartiles of intake.

The relative rank for FV consumption was used to define tracking and the dynamics of FV intake. Four patterns were defined: tracking high, tracking low, decreased and increased. Tracking was defined as being in the same relative position in both 2008 and 2011 follow-ups. If an adolescent remained in the lowest FV intake quartiles over the 3-year period, this was defined as ‘tracking low’; if an adolescent remained in the upper ones this was defined as ‘tracking high’. The dynamics of FV consumption were examined by identifying the downward or upward changes over the period from adolescents who moved to the opposite quartile. In the case that an adolescent who was originally in the lowest quartile moved to the highest one, this was defined as ‘increased’; when an adolescent moved from the highest quartile to the lowest one, this was defined as ‘decreased’.

### Independent variables

Independent variables included were sex and socio-economic status (SES) at the 2008 follow-up. SES was measured by tertiles of an assets index that was built based on factorial analysis considering nineteen house assets and schooling of the family’s head (0–4, 5–8, 9–11, 12 or more years). The assets originally included in the questionnaire were those collected in standardized socio-economic position classification systems in Brazil.

### Statistical analyses

The median daily frequency of FV consumption along with the interquartile range (25th–75th percentile) at the two follow-up visits was calculated due to its asymmetric distribution. To evaluate if there was a significant change in FV intake between both surveys, the Wilcoxon signed-rank test was performed. *P*<0·05 was considered significant.

The stability of FV consumption was studied through different approaches. First, the overall proportion of participants who maintained the same relative position at any level over time was assessed. The proportion of stability by chance between two time points would be 25 % assuming that participants could move randomly to any of the other quartiles at time 2. The sex differences were assessed using a *χ* ^2^ test. Furthermore, we calculated the weighted kappa coefficient (*κ*_w_) to test the agreement between each individual’s relative positions in quartiles in both waves. Note that *κ*_w_=0 when the observed agreement equals that expected by chance and *κ*_w_=1 when the tracking is perfect^(^ ^33^ ^)^. A cut-off for the *κ*_w_ statistic was a value >0·2 to indicate the existence of tracking, >0·4 for moderate tracking and >0·8 for excellent tracking^(^ ^21^ ^–^ ^24^ ^)^.

Finally, the proportions of participants who remained in the highest or lowest quartile (tracking high or tracking low) and those who moved to the opposite one (decreased or increased) over the 3-year period were calculated stratified by tertiles of SES^(^ ^20^ ^)^.

The proportions of tracking and changes were adjusted for adolescents’ baseline leisure-time physical activity (minutes per day), occupation (hours per day) and schooling (attended school during the last year) using Poisson regression with robust variance^(^ ^34^ ^)^. All the analyses were stratified by sex and assets index.

Data analyses were performed using the STATA statistical software package version 12·1.

The study protocol was approved by the Research Ethics Committee of the School of Medicine, Federal University in Pelotas and written informed consent was obtained from all participants at each visit.

## Results

A total of 3915 adolescents (52 % female) who participated in both waves were included in the analyses. The mean age at 2008 follow-up was 14·7 (sd 0·30) years and 18·4 (sd 0·25) years at the 2011 follow-up. In 2008, the median leisure-time physical activity was 180 min/d, 98 % attended school during the previous year and approximately 20 % of the adolescents had a job, more than 80 % of whom had a part-time job.

**Fig. 1:**
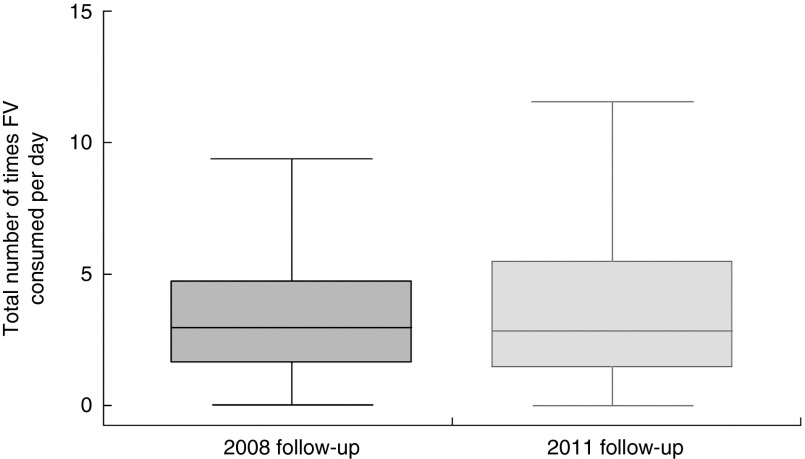
Adolescents’ daily frequency of fruit and vegetable (FV) intake at 15-year (2008) and 18-year (2011) follow-ups; 1993 Pelotas Birth Cohort, Rio Grande do Sul, Brazil, 2008–2011/12 (*n* 3915). Data are presented as box-and-whisker plots (excluding outliers) where the bottom and top of the box represent the 25th and 75th percentile respectively (i.e. the interquartile range), the line within the box represents the median, and the bottom and top whisker represent the minimum and maximum value, respectively

The distribution of the total number of times FV were consumed daily at 2008 and 2011 follow-ups is shown in Fig. 1.

In terms of FV consumption medians, a decrease was observed for the entire sample from 2008 to 2011: the median total number of times of FV consumption per day was 2·97 and 2·86, respectively (*P*<0·001; data not shown). Specifically, in 2011 adolescents reported consuming FV less frequently than in 2008, although the differences were not significant except for the girls in the lowest tertile of the assets index, who increased their median daily FV consumption frequency by approximately 25 % (Table 1).

**Table 1:** Adolescents’ median frequency of FV intake* at 15-year (2008) and 18-year (2011) follow-ups stratified by sex and assets index; 1993 Pelotas Birth Cohort, Rio Grande do Sul, Brazil, 2008–2011/12 (*n* 3915)

Assets index	2008 follow-up		2011 follow-up		Change†,‡
(tertile)	Median	IQR	Median	IQR	(%)
Boys					
1 (lowest)	3·08	1·75–4·93	3·01	1·61–5·78	2·27
2	2·86	1·69–4·77	2·57	1·31–5·12	3·34
3 (upper)	2·66	1·29–4·60	2·37	1·18–4·60	10·90
Girls					
1 (lowest)	2·95	1·63–4·57	3·70	1·87–7·55	25·42
2	3·04	1·83–4·76	2·79	1·56–5·66	8·22
3 (upper)	3·00	1·68–4·90	2·65	1·47–4·70	11·67

FV, fruits and vegetables; IQR, interquartile range.

* Total number of times FV consumed per day.

† The change is positive only for girls in the lowest tertile of assets index.

‡ Only the difference between FV intake medians for girls in the lowest tertile of assets index was significant, *P*<0·001 (Wilcoxon signed-rank test).

### Proportion of tracking and changing quartile rank positions over a 3-year period

The tracking tendency of the cohort was first examined by determining to what extent participants maintained their relative positions at any level of intake (Table 2). Approximately 40 % of boys and girls belonging to the upper tertile of assets index, and about 30 % of those belonging to the lowest one, maintained their quartile positions 3 years later. The *κ*_w_ values for FV intake indicated the presence of tracking (*κ*_w_>0·2) for adolescents of both sexes of higher SES.

**Table 2:** Tracking patterns* of adolescents’ FV intake† between 15- and 18-year follow-ups stratified by sex and assets index; 1993 Pelotas Birth Cohort, Rio Grande do Sul, Brazil, 2008–2011/12 (*n* 3915)

	Boys			Girls		
Assets index	Overall tracking‡			Overall tracking‡		
(tertile)	%	95 % CI	*κ*_w_ §	%	95 % CI	*κ*_w_ §
1 (lowest)	30·2	26·7, 34·2	0·14	30·8	27·5, 34·4	0·13
2	35·7	32·1, 39·6	0·24	35·4	32·0, 39·3	0·23
3 (upper)	38·6	35·0, 42·7	0·30	40·5	36·9, 44·4	0·32

FV, fruits and vegetables; *κ*_w_, weighted kappa.

* Tracking is adjusted for adolescents’ leisure-time physical activity, occupation and schooling at 15-year follow-up.

† Total number of times FV consumed per day.

‡ Proportion of adolescents who remained in the same quartile in both 2008 and 2011 follow-ups at any level.

§ *κ*_w_ coefficients measure the agreement based on quartile position; *κ*_w_>0·2 suggests tracking.

**Fig. 2:**
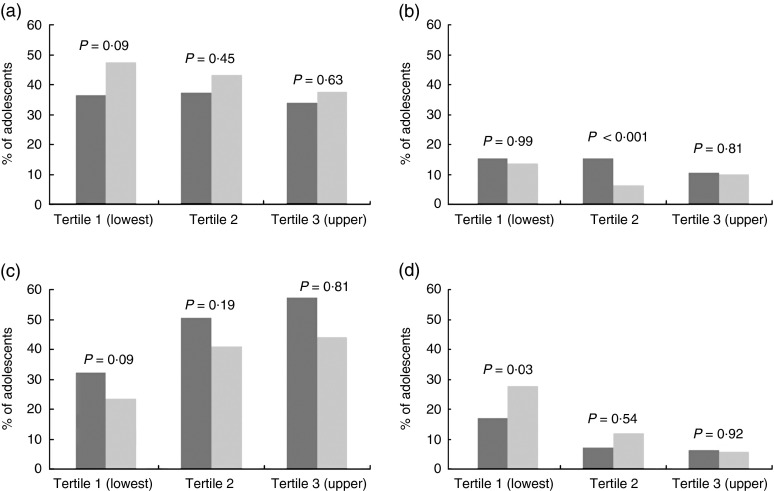
Tracking (a, tracking high; c, tracking low) and changes (b, decreased; d, increased) in adolescents’ (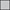, girls; 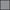, boys) daily frequency of fruit and vegetable intake from 15 to 18 years according to tertiles of the assets index; 1993 Pelotas Birth Cohort, Rio Grande do Sul, Brazil, 2008–2011/12 (*n* 3915). *P* values are from 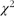
\chi^2 tests of sex differences

The proportions of adolescents in the highest and lowest quartiles of FV intake at baseline who remained in those same quartiles or moved to the opposite quartile are shown in Fig. 2. Almost half of the girls of the lowest tertile of assets index and 37 % of girls in the upper one presented a tracking of high consumption of FV. Instead, the proportion of boys remaining in the highest quartile of FV intake across time was nearly the same for each tertile of assets index. Among the adolescents of the upper assets index tertile, almost 60 % of boys and about 45 % of girls remained in the lowest quartile of FV intake.

The proportion of adolescents who increased their intake of FV differed according to SES and sex. About 6 % of girls and boys belonging to the upper tertile of assets index who initially were in the lowest quartile of FV intake moved to the highest one over the period between follow-ups. Among those belonging to the lowest SES, approximately one-third of girls and 20 % of the boys showed this positive shift in FV intake.

We also performed crude analyses to compare with the adjusted ones. Although some differences were found in the proportions of tracking and changes, in general the results showed the same pattern.

## Discussion

We have described the stability and changes in FV consumption over a 3-year period during adolescence. Two main conclusions emerge. First, adolescents taking part in the 1993 Pelotas Birth Cohort showed a slight decrease in their daily frequency of FV consumption during the study period. Second, there was presence of tracking (maintenance in the same quartile over time) which was evidenced by the percentage of agreement between quartiles and *κ*_w_ coefficients.

We expected that FV intake would be positively associated with SES. However, our results indicated that adolescents belonging to the upper tertile of assets index reduced their daily frequency more than those who were in the lowest tertile over a 3-year period. These findings are consistent with the existing literature. Some studies observed a decline in the quality of adolescents’ overall pattern of dietary intake^(^ ^25^ ^,^ ^35^ ^)^. At this age, the influence of parents and the home environment on diet decreases. A study from Norway reported an inverse correlation between the amount of money spent on sweets and snacks, and consumption of dietary fibre, indicating that less healthy foods could replace healthier ones when adolescents buy their own food^(^ ^36^ ^)^. In a longitudinal study about dietary intake patterns from childhood into adolescence in China, Wang *et al*. showed that children belonging to families of higher education were more likely to have a high-fat diet but less likely to maintain a high level of FV intake, regardless of family income^(^ ^24^ ^)^. In a review about tracking of dietary patterns from childhood to adolescence, Madruga *et al.* concluded that dietary patterns in childhood may persist until adolescence, although such patterns may be changed or discontinued throughout adolescence^(^ ^37^ ^)^. In addition, these results are in concordance with the findings of our analyses, which showed that more than half of boys and girls of the highest SES remained in the lowest quartile of FV consumption over 3 years during adolescence. It is possible that these adolescents, whose families were classified in the upper SES, replaced healthy foods like FV with snacks and sweets, a phenomenon that is becoming more prevalent among adolescents and young adults^(^ ^38^ ^)^. We also observed an increased FV intake in girls from low SES.

The analyses were adjusted for leisure-time physical activity, occupation and schooling. Some characteristics such as occupation, employment or schooling may modify dietary behaviours. Additionally, physical activity is associated with FV intake in adolescents^(^ ^39^ ^,^ ^40^ ^)^. Still, the fact that adjusted analyses showed the same pattern as the crude ones may indicate the presence of a more complex scenario affecting adolescents’ intake habits. A review has identified FV preferences and availability as the most consistent influences on adolescents’ FV intake^(^ ^41^ ^)^. Thompson *et al*. reported that attitudes about healthy diets were more important than knowledge about a healthy diet and sociodemographic characteristics in adolescents and adults^(^ ^42^ ^)^.

In terms of tracking, the proportion of adolescents who remained in the same quartile (at any level) across the study period (30–40 %) exceeded the proportion expected by chance (25 %), thus indicating stability^(^ ^19^ ^)^. These results are in concordance with evidence from different countries that suggests existence of low to moderate tracking despite the differences in dietary assessment, age and population groups, and follow-up duration^(^ ^15^ ^,^ ^21^ ^,^ ^23^ ^,^ ^24^ ^,^ ^26^ ^)^. For example, Wang *et al*. also revealed that 34 % of students remained in the same rank position over a 6-year period^(^ ^24^ ^)^. A study conducted by Li and Wang showed approximately 40 % of overall tracking over 1 year in urban low-income African-American adolescents^(^ ^21^ ^)^. Further comparisons can be difficult due to the variety of methods used to assess tracking^(^ ^19^ ^)^.

We observed that girls tended to remain in the highest quartile of FV intake more than boys. This finding is in line with the literature reporting that women above 15 years of age tend to be more concerned about food and nutrition, to be more health conscious and to manifest beneficial changes in food behaviours better than men^(^ ^43^ ^)^.

The main limitation of the present study is the difference in dietary assessment methodologies at both follow-up measurements. As explained in the ‘Methods’ section, the 15-year follow-up FFQ provided open frequency categories, while the 18-year follow-up questionnaire presented closed ranges of responses, thus reducing the comparability between them. However, the 18-year follow-up FFQ categories were provided in a wide-ranging format without gaps (i.e. ‘never or less than one a month’, ‘one to three times a month’, ‘once a week’, ‘two to four times a week’, ‘five to six times a week’, ‘one time a day’, ‘two to four times a day’ and ‘five or more times a day’), which reduced the possibility of incomplete responses due to lack of options^(^ ^44^ ^)^. Consequently, the frequency intake possibilities of responses were the same in both methodologies, with a low possibility of bias. Furthermore, both questionnaires had similarities concerning some issues that influence the participants’ responses, such as the period of recall time, list and order of FV items, and also the order of the response categories (response keys were ordered by increasing intake)^(^ ^45^ ^)^.

Moreover, the way the FV intake is calculated tends to overestimate the real consumption and FV are often over-reported, particularly if each item is listed singly in a long list^(^ ^44^ ^)^.

Our data set may be subject to the inaccuracies inherent to the reporting of dietary intake. Nevertheless, the FFQ is the dietary assessment method indicated for epidemiological studies, particularly for large-scale samples^(^ ^46^ ^,^ ^47^ ^)^, and it is appropriate for evaluating adolescents’ usual eating habits^(^ ^48^ ^)^.

Perhaps some of the findings were affected by statistical properties. First, when groups are arbitrarily defined based on the distribution of values (i.e. quartiles), the results of the tracking analysis depend on the arbitrary choice for the division of subgroups. Second, participants can change substantially within their original percentile without influencing the tracking, while a minor shift at the border of two quartiles actually will influence the tracking^(^ ^19^ ^)^. Third, those belonging to the extremes are more likely to keep their ranking position than others^(^ ^49^ ^)^. However, the analyses we performed (percentage agreement between quartiles and *κ*_w_ coefficients) to assess the FV intake patterns have provided consistent results, confirming that our findings are robust enough to make assumptions about the stability of FV intake in the sample.

Strengths of the present study include its prospective design, the large and population-based sample and the follow-up rates, which are high for studies in low- and middle-income countries^(^ ^50^ ^)^.

From the public health and educational standpoint, our findings highlight the importance of interventions to promote healthy behaviours and to prevent the risk of non-communicable diseases in adolescents. From a chronic disease perspective, the tracking analyses are of particular concern for individuals with extremes of consumption (i.e. very low fibre consumption)^(^ ^51^ ^)^. In our study we found that adolescents with low levels of intake tended to maintain those levels over time. This persistence possibly will have an impact on the development of nutrition-related chronic diseases. Therefore, it is of concern to be aware of adolescents’ eating behaviours.

The nutritional and epidemiological transition in low- and middle-income countries including Brazil is going towards an unfavourable direction^(^ ^52^ ^)^. For example, our sample is characterized by an important proportion of overweight, 27·6 % and 27·3 % in the 15- and 18-years follow-ups, respectively (data not shown). It seems that adolescents in our cohort are having less healthy choices and consequently greater exposure to risk factors for non-communicable chronic diseases.

To our knowledge, the present study is the first one from a low- to middle-income country to examine the stability and changes in FV intake with longitudinal data. Furthermore, no earlier study has analysed tracking of FV intake comparing adolescents from different SES; thus these findings are interesting in assessing the role of socio-economic factors on this issue. Our data showed that FV consumption tracks moderately across 3 years during adolescence, especially in those with higher SES. It will be interesting to see what will happen in the next few years.

Taking all this together, we can conclude that regardless of the statistical analysis, the patterns of FV intake over time are complex to understand. Yet, we encourage future research to consider the influence of dietary behaviours and statistical factors in the tracking phenomena in order to identify whether the FV consumption remains stable or changes across the entire life course.
